# Biomarker-based prediction of progression in MCI: Comparison of AD signature and hippocampal volume with spinal fluid amyloid-β and tau

**DOI:** 10.3389/fnagi.2013.00055

**Published:** 2013-10-11

**Authors:** Bradford C. Dickerson, David A. Wolk, Alzheimer's Disease Neuroimaging Initiative

**Affiliations:** ^1^Frontotemporal Dementia Unit, Massachusetts General Hospital and Harvard Medical SchoolBoston, MA, USA; ^2^Department of Neurology, Massachusetts General Hospital and Harvard Medical SchoolBoston, MA, USA; ^3^Massachusetts Alzheimer's Disease Research Center, Massachusetts General Hospital and Harvard Medical SchoolBoston, MA, USA; ^4^Athinoula A. Martinos Center for Biomedical Imaging, Massachusetts General Hospital and Harvard Medical SchoolBoston, MA, USA; ^5^Department of Neurology, University of PennsylvaniaPhiladelphia, PA, USA; ^6^Alzheimer's Disease Core Center, University of PennsylvaniaPhiladelphia, PA, USA; ^7^Penn Memory Center, University of PennsylvaniaPhiladelphia, PA, USA

**Keywords:** Alzheimer's disease, MRI, biomarkers, mild cognitive impairment, CSF biomarkers

## Abstract

**Objective:** New diagnostic criteria for mild cognitive impairment (MCI) due to Alzheimer's disease (AD) have been developed using biomarkers aiming to establish whether the clinical syndrome is likely due to underlying AD. We investigated the utility of magnetic resonance imaging (MRI) and cerebrospinal fluid (CSF) biomarkers in predicting progression from amnesic MCI to dementia, testing the hypotheses that (1) markers of amyloid and neurodegeneration provide distinct and complementary prognostic information over different time intervals, and that (2) evidence of neurodegeneration in amyloid-negative MCI individuals would be useful prognostically.

**Methods:** Data were obtained from the ADNI-1 (Alzheimer's Disease Neuroimaging Initiative Phase 1) database on all individuals with a baseline diagnosis of MCI, baseline MRI and CSF data, and at least one follow-up visit. MRI data were processed using a published set of *a priori* regions of interest to derive a measure known as the ``AD signature,'' as well as hippocampal volume. The CSF biomarkers amyloid-β, total tau, and phospho tau were also examined. We performed logistic regression analyses to identify the best baseline biomarker predictors of progression to dementia over 1 or 3 years, and Cox regression models to test the utility of these markers for predicting time-to-dementia.

**Results:** For prediction of dementia in MCI, the AD signature cortical thickness biomarker performed better than hippocampal volume. Although CSF tau measures were better than CSF amyloid-β at predicting dementia within 1 year, the AD signature was better than all CSF measures at prediction over this relatively short-term interval. CSF amyloid-β was superior to tau and AD signature at predicting dementia over 3 years. When CSF amyloid-β was dichotomized using previously published cutoff values and treated as a categorical variable, a multivariate stepwise Cox regression model indicated that both the AD signature MRI marker and the categorical CSF amyloid-β marker were useful in predicting time-to-event diagnosis of AD dementia.

**Conclusion:** In amnesic MCI, short-term (1 year) prognosis of progression to dementia relates strongly to baseline markers of neurodegeneration, with the AD signature MRI biomarker of cortical thickness performing the best among MRI and CSF markers studied here. Longer-term (3 year) prognosis in these individuals was better predicted by a marker indicative of brain amyloid. Prediction of time-to-event in a survival model was predicted by the combination of these biomarkers. These results provide further support for emerging models of the temporal relationship of pathophysiologic events in AD and demonstrate the utility of these biomarkers at the prodromal stage of the illness.

## INTRODUCTION

When insidious in onset and gradually progressive, mild cognitive impairment (MCI) is a clinical syndrome commonly arising as a result of neurodegenerative pathology (Petersen et al., 2006). In living persons, evidence of neurodegenerative pathology is provided by a growing array of imaging and fluid biomarkers. If the goal is to determine whether MCI appears highly likely to be due to underlying Alzheimer pathology, the recently published MCI diagnostic criteria require evidence of (1) cerebral amyloidosis [amyloid positron emission tomography (PET) or cerebrospinal fluid (CSF) amyloid-β] and (2) neurodegeneration [magnetic resonance imaging (MRI)-derived atrophy, fluorodeoxyglucose (FDG)-PET-derived hypometabolism, or CSF tau; Albert et al., 2011]. A number of studies have now shown that, within a group of persons with MCI, the presence and prominence of these biomarkers are predictive of the likelihood of Alzheimer’s disease (AD) dementia within a few years (Jack et al., 1999; Hansson et al., 2006; Wang et al., 2006; Vemuri et al., 2009b; Visser et al., 2009; Blennow et al., 2010; De Meyer et al., 2010; Jack et al., 2010; Landau et al., 2010; Buchhave et al., 2012). Despite the importance of observations from these studies, a number of questions remain, particularly when considering how to use biomarkers in the design of clinical trials of putative interventions. Further, as more clinicians are beginning to incorporate these measures into clinical practice, a deeper understanding of the relative implications of these biomarkers is critical.

In persons with MCI, what are the best MRI-derived biomarkers of neurodegeneration with regard to prediction of progression to dementia? One very commonly used measure is hippocampal volume, which has consistently been shown to predict dementia in MCI (Frisoni et al., 2010). We have developed an AD signature cortical thickness marker (Dickerson et al., 2009), and hypothesize that this marker will outperform commonly used MRI-derived biomarkers as an indicator of AD-related neurodegeneration in MCI that is predictive of AD dementia.

Another major question relates to the temporal utility of biomarkers. What are the best markers for short-term vs. longer-term prediction of dementia? Although current clinico-pathologic constructs of AD require evidence of cerebral amyloidosis, data are conflicting as to whether markers of amyloid or neurodegeneration best predict dementia and to our knowledge none have specifically tested hypotheses about the comparative utility of amyloid vs. neurodegenerative markers at different time intervals. We tested two hypothesis here: (1) rapid progression (i.e., over 1 year) from MCI to AD dementia is better predicted by markers of neurodegeneration rather than the presence of amyloid; (2) longer-term progression from MCI to dementia (i.e., 3 years) is best predicted by the presence of abnormal levels of brain amyloid. This prediction follows from the notion that cerebral amyloidosis may be a relatively earlier development in AD pathophysiology compared to evidence of neurodegeneration measured using *in vivo* methods (Jack et al., 2013). Further, neurodegenerative markers appear to be more sensitive to disease state than measures of cerebral amyloid (Jack et al., 2009; Vemuri et al., 2009a). As such, amyloid measures may differentiate individuals who will eventually progress to AD over longer-term follow-up while neurodegenerative markers may indicate an elevated risk for more proximate cognitive decline and dementia.

Finally, the focus of a number of studies of biomarker prediction of AD dementia in amnestic MCI has been on the 50–75% of subjects with evidence of cerebral amyloidosis. What about the other individuals, especially those who may show evidence suggestive of neurodegeneration (Knopman et al., 2012; Petersen et al., 2013)? Are MRI-derived markers useful in predicting dementia in individuals with MCI who do not have evidence of cerebral amyloidosis?

Here we undertook a set of analyses of the Alzheimer’s Disease Neuroimaging Initiative (ADNI) dataset to investigate these questions, focusing on the utility of MRI and CSF biomarkers for prognosis in MCI.

## MATERIALS AND METHODS

### PARTICIPANTS

Data used in the preparation of this article were obtained from the ADNI database^1^. The ADNI was launched in 2003 by the National Institute on Aging (NIA), the National Institute of Biomedical Imaging and Bioengineering (NIBIB), the Food and Drug Administration (FDA), private pharmaceutical companies, and non-profit organizations, as a $60 million, 5-year public–private partnership. The primary goal of ADNI has been to test whether serial MRI, PET, other biological markers, and clinical and neuropsychological assessment can be combined to measure the progression of MCI and early AD. Determination of sensitive and specific markers of very early AD progression is intended to aid researchers and clinicians to develop new treatments and monitor their effectiveness, as well as lessen the time and cost of clinical trials.

The Principal Investigator of this initiative is Michael W. Weiner, MD, VA Medical Center and University of California – San Francisco. ADNI is the result of efforts of many co-investigators from a broad range of academic institutions and private corporations, and subjects have been recruited from over 50 sites across the U.S. and Canada. The initial goal of ADNI was to recruit 800 subjects but ADNI has been followed by ADNI-GO and ADNI-2. To date these three protocols have recruited over 1500 adults, ages 55–90, to participate in the research, consisting of cognitively normal older individuals, people with early or late MCI, and people with early AD. The follow-up duration of each group is specified in the protocols for ADNI-1, ADNI-2, and ADNI-GO. Subjects originally recruited for ADNI-1 and ADNI-GO had the option to be followed in ADNI-2. For up-to-date information, see www.adni-info.org.

For the current analysis, we selected individuals with a baseline diagnosis of MCI who had baseline MRI and CSF data available, and at least 1 year of clinical follow-up (*n* = 154). Detailed diagnostic, inclusion, and exclusion criteria are described on the ADNI website^2^.

### STANDARD PROTOCOL APPROVALS, REGISTRATIONS, AND PATIENT CONSENTS

Each participant gave written informed consent in accordance with institutional Human Subjects Research Committee guidelines.

### MRI IMAGING AND ANALYSIS

Magnetic resonance imaging scans were collected on a 1.5T scanner using a standardized MPRAGE protocol: sagittal plane, TR/TE/TI, 2400/3/1000 ms, flip angle 8°, 24 cm FOV, 192 × 192 in-plane matrix, 1.2 mm slice thickness (Jack et al., 2008). Fully pre-processed scans were downloaded for analysis.

**FIGURE 1:**
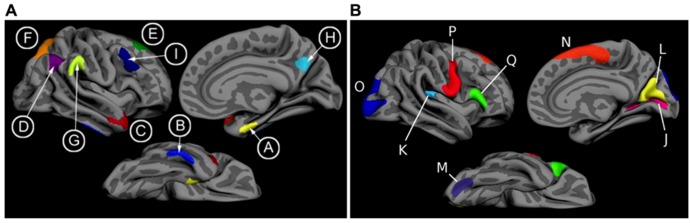
**(A)** The cortical signature of AD is composed of *a priori* regions of interest in which consistent atrophy has been previously observed in multiple samples of patients with mild AD dementia. **(B)** The cortical signature of normal aging is composed of *a priori* regions of interest in which consistent atrophy has been previously described in healthy cognitively intact older adults compared with younger adults. We calculated the “AD signature index” measure by performing a linear regression with the Aging signature (excluding regions overlapping with AD signature regions; see Figures 1 and 2 of Bakkour et al., 2013) as the independent variable and the AD signature as the dependent variable. The residuals of this regression analysis were then saved as the “AD signature index.” Key: A: medial temporal, B: inferior temporal, C: temporal pole, D: Angular, E: superior frontal, F: superior parietal, G: supramarginal, H: precuneus, I: middle frontal, J: calcarine, K: caudal insula, L: cuneus, M: caudal fusiform, N: dorsomedial frontal, O: lateral occipital, P: precentral, Q: inferior frontal.

T1 image volumes were examined quantitatively by a cortical surface-based reconstruction and analysis of cortical thickness, using a hypothesis-driven approach as described in multiple previous publications (Bakkour et al., 2009; Dickerson et al., 2009, 2011; Wolk et al., 2010). Briefly, we utilized nine regions of interest (ROIs, see **Figure 1**) previously determined to be associated with AD, the “cortical signature” of AD (Bakkour et al., 2009; Dickerson et al., 2009).

For the purposes of this study, we employed a primary diagnostic biomarker, the single summary “AD signature measure,” the average thickness of all nine ROIs. With the goal of adjusting this measure for normal age-related influences on these brain regions, we also measured a set of “Aging signature” ROIs, as previously published (Bakkour et al., 2013). We calculated an “AD signature index” measure by first performing a linear regression in the amyloid-negative control group with the Aging signature as the independent variable and the AD signature as the dependent variable. We then used this equation to calculate the “AD signature index values for each MCI patient.” Thus, an individual with a lower AD signature index value has cortical thickness within the AD signature ROIs that is disproportionately smaller than the thickness of the Aging signature ROIs, likely reflecting more specific AD-related neurodegeneration. Alternatively, an individual with a higher AD signature index value has cortical thickness within the AD signature ROIs that is of similar relative magnitude to Aging signature ROIs, possibly reflecting more diffuse effects.

In addition, for comparison purposes, we analyzed hippocampal volume using the measure provided by the automated segmentation procedure from FreeSurfer, divided by total intracranial volume. Our standard procedure is to visually inspect selected coronal slices of each automated segmentation and identify scans with errors in processing of the structure of interest. We also inspect the distribution of the quantitative volumetric data and review scans at either tail of the distribution and outliers in greater detail. In the present analysis, no scans were identified with important errors of hippocampal segmentation.

### BASELINE CEREBROSPINAL FLUID MEASURES

We also examined baseline CSF levels of amyloid-β, total tau (t-tau), and phosphorylated tau (p-tau). For the primary analyses, we used the raw values as continuous measures; however, t-tau and p-tau were log-transformed to better approximate normality in distribution. For analyses in which we classified subjects as having CSF amyloid-β values consistent with those of autopsy-proven AD, we used a cutoff value of levels less than 192 (Shaw et al., 2009). Individuals with levels ≥geq192 were considered to be unlikely to have cerebral amyloidosis.

### LONGITUDINAL OUTCOMES

Here we used outcomes at 1 or 3 years. The primary outcome measures used in the present analysis were conversion to a diagnosis of AD dementia at 1 or at 3 years.

### STATISTICAL ANALYSIS

Tests of group differences were performed using Chi-square analysis (for frequencies) or Analysis of Variance (for continuous measures) with *post hoc* pairwise comparisons where relevant; *a* = 0.05. Since effect sizes were expected to be subtle and strong *a priori* hypotheses were being tested, no multiple comparisons correction procedures were performed. In addition, the impact of biomarkers on clinical outcome was analyzed using separate logistic regression models for each of the two intervals of follow-up, constructed using the dichotomous conversion to dementia outcome measure as the dependent variable. Cox regression models were constructed to investigate the relationship of baseline biomarkers to the likelihood of progression to AD dementia using a more fine-grained time-to-event outcome rather than the two follow-up intervals employed in the other analyses. A multivariate Cox regression model was then constructed including independent variables that reached a trend-level effect (*p* < 0.1) in the univariate analyses (*p*-value-to-enter <0.05). Covariates of age, education, and gender were generally not significant in the models and had minimal impact on the findings. Statistical analyses were performed using IBM SPSS 21.0.

## RESULTS

Of the 156 MCI participants with baseline MRI and CSF data who were followed for 1 year, 31 (20%) were diagnosed with probable AD dementia. Of the 111 who had 3-year outcome data, 48 (43%) were diagnosed with probable AD dementia. In the subset of MCI participants with baseline CSF evidence of cerebral amyloidosis, 26 of 116 (22%) were diagnosed with probable AD dementia at 1 year and 45 of 83 (54%) at 3 years. In contrast, in the subgroup of MCI participants with normal baseline CSF amyloid-β levels, only 5 of 40 (13%) converted to AD dementia at 1 year and 3 of 27 (11%) at 3 years. See **Table 1** for additional details.

**Table 1:** Demographic and baseline biomarker characteristics of sample.

Subject group	1-year outcome (*N* = 156)		3-year outcome (*N* = 111)	
N (%) or M (SD)	MCI (*N* = 125)	AD (*N* = 31)	MCI (*N* = 63)	AD (*N* = 48)
Age (years)	74.9 (7.6)	72.3 (6.90)	74.7 (7.3)	74.3 (7.7)
Gender	84 M: 41 F	17 M: 14 F	47 M: 16 F	30 M: 18 F
Education (years)	15.8 (3.0)	15.1 (3.2)	15.6 (3.0)	15.6 (3.4)
MMSE	27.5 (1.7)	26.7 (1.9)	27.3 (1.8)	26.7 (1.9)
CDR-SB	1.9 (0.8)	2.4 (0.9)^*^	1.7 (0.6)	2.2 (1.0)^*^
CSF amyloid-β Z score	-0.73 (1.00)	-0.99 (0.70)	-0.56 (1.12)	-1.15 (0.65)^**^
CSF Total tau Z score	0.69 (1.07)	1.17 (1.13)	0.74 (1.22)	1.26 (1.13)^*^
CSF P-tau Z score	0.69 (1.06)	1.20 (0.99)^*^	0.62 (1.04)	1.12 (0.93)^*^
AD signature Z score	-0.82 (1.13)	-1.82 (1.27)^**^	-0.63 (1.12)	-1.26 (1.19)^*^
Hippo vol Z	-0.94 (1.14)	-1.24 (1.06)	-0.75 (1.05)	-1.15 (1.04)^†^

* *p* < 0.05,

** *p* < 0.005 groups are different from each other.

† *p* = 0.05, groups demonstrate trend-level difference from each other.

We first sought to determine which of the baseline biomarkers would be useful in prediction of the likelihood of a diagnosis of probable AD dementia in the entire sample of MCI subjects. For the 1-year outcome interval, baseline cortical thickness measured with the AD signature MRI biomarker index was strongly associated with the likelihood of probable AD: a logistic regression model predicting a 1-year AD outcome indicated a nearly threefold increase in the likelihood of AD dementia for each 1 SD thinner cortex [odds ratio (OR) = 2.7, 95% C.I.: [1.7–4.5], *p* < 0.0001). In addition, baseline CSF p-tau levels were predictive of AD dementia, with each 1 SD increase in CSF p-tau levels being associated with a 1.7-fold increase in the likelihood of AD dementia (OR = 1.7, 95% C.I.: [1.09–2.7], *p* = 0.02). None of the other biomarkers demonstrated effects or trend-level effects. In the stepwise multivariate logistic regression model, the AD signature MRI marker entered but CSF p-tau did not, indicating that CSF p-tau did not explain additional variance in outcome beyond that explained by the AD signature MRI marker.

In contrast, 3-year conversion was best predicted by baseline CSF amyloid-β levels, with each 1 SD of reduction indicating a 1.9-fold increase in 3-year likelihood of AD dementia (OR = 1.9, 95% C.I.: [1.2–3.0], *p* = 0.003). A slightly weaker effect was observed for the AD signature index (OR = 1.7, 95% C.I.: [1.12–2.6], *p* = 0.01). Significant effects were also observed for CSF p-tau (OR = 1.8, 95% C.I.: [1.14–2.7], *p* = 0.01) and CSF t-tau (OR = 1.6, 95% C.I.: [1.06–2.5], *p* = 0.03) while hippocampal volume displayed a strong trend (OR = 1.5, 95% C.I.: [1.00–2.29], *p* = 0.05). In the stepwise multivariate model, CSF amyloid-β entered but the other two did not.

**FIGURE 2:**
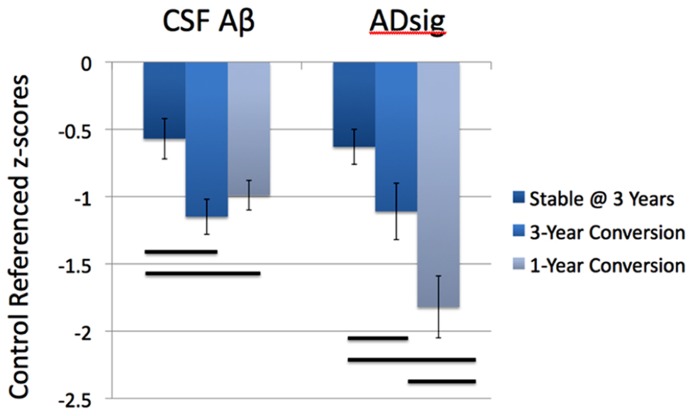
**Values for the AD signature cortical thickness and CSF amyloid-β for each of the three MCI subgroups based on outcome (stable over 3 years, 3-year converters, and 1-year converters).** The mean values for CSF amyloid-β are lower in both groups of converters than in stable MCI (left), while the values for AD signature index of cortical thickness are lower in the 1-year converters than in the other two groups (right; all values shown are Z scores derived from the normative values of controls and bars represent statistically significant comparisons).

**Figure 2** depicts the values for the AD signature cortical thickness MRI marker and CSF amyloid-β for each of the three MCI subgroups based on outcome (stable over 3 years, 3-year converters who did not convert by year 1, and 1-year converters). The mean values for CSF amyloid-β are lower in both groups of converters than in stable MCI (1-year: *p* < 0.05; 3-year: *p* < 0.01), but there is no difference based on year of conversion (*p* > 0.3). Alternatively, values for AD signature cortical thickness are lower for both groups of converters than the stable group (1-year: *p* < 0.001; 3-year: *p* = 0.05), but also for the 1-year converters compared to the 3-year converters (*p* < 0.05; all values shown are Z scores derived from the normative values of controls).

We next investigated the utility of biomarkers for prediction of a diagnosis of AD dementia in subgroups of MCI subjects divided on the basis of baseline CSF amyloid-β levels. In the subgroup of MCI subjects with abnormally low baseline CSF amyloid-β levels (consistent with cerebral amyloidosis), 1-year conversion to AD dementia was predicted by the AD signature MRI biomarker (OR = 2.2, 95% C.I.: [1.3–3.8], *p* = 0.005). None of the other biomarkers were predictive in these univariate models. For 3-year prediction, a significant effect for the AD signature MRI biomarker (OR = 1.7, 95% C.I.: [1.1–2.7], *p* = 0.03) and a trend for hippocampal volume (OR = 1.5, 95% C.I.: [0.95–2.5], *p* = 0.08) were observed.

In the subgroup of MCI subjects with normal CSF amyloid-β levels, indicating the likely absence of cerebral amyloidosis, 1-year conversion to AD dementia was best predicted by the AD signature MRI biomarker (OR = 6.4, 95% C.I.: [1.5–27.5], *p* = 0.01), with hippocampal volume showing utility as well (OR = 3.5, 95% C.I.: [1.2–10.7], *p* = 0.03) but not entering the multivariate model. None of the CSF markers demonstrated predictive value. For 3-year prediction, none of the markers were useful although power was extremely low due to the small number of individuals who were diagnosed with AD dementia.

**FIGURE 3:**
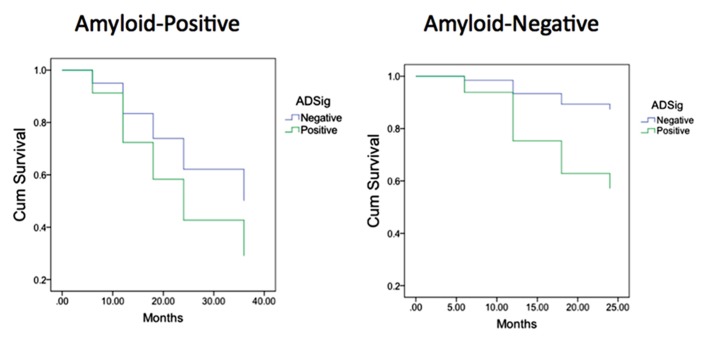
**Survival curves in MCI participants who were “amyloid-negative” at baseline (left) vs. those who were “amyloid positive” (right) as a function of baseline AD signature index using dichotomous cutoff**.

Finally, we performed a survival analysis to investigate the utility of these biomarkers for predicting the time to a diagnosis of AD dementia. Univariate Cox proportional hazards regression models indicated that each of the biomarkers was a predictor of time to diagnosis of AD dementia over the 3-year follow-up period (**Table 2**). In multivariate analysis, a stepwise forward conditional model demonstrated that the AD signature MRI biomarker was the best and only predictor when each independent variable was entered as a continuous variable. However, when CSF amyloid-β was dichotomized using previously published cutoff values (Shaw et al., 2009) and treated as a categorical variable, the multivariate stepwise Cox regression model indicated that both the AD signature MRI marker and the categorical CSF amyloid-β marker were useful in predicting time-to-event diagnosis of AD dementia. Of all the models, this was the model with the overall strongest statistical results (*X*^2^ = 19.4, *p* < 0.001). This result is illustrated in **Figure 3** in which the AD signature was also dichotomized to maximize sensitivity and specificity between amyloid-negative controls and amyloid-positive mild AD patients from the ADNI cohort.

**Table 2:** Results of Cox regression analyses of baseline CSF and MRI biomarker measures predicting probable AD diagnosis.

	*X*^2^	HR	95% CI
AD signature	13.7 (*p* < 0.001)^**^	1.61	1.25–2.08
AD signature dichotomous	12.9 (*p* < 0.001)^**^	2.28	1.44–3.63
CSF amyloid-β dichotomous	12.2 (*p* < 0.001)^**^	3.66	1.68–7.99
CSF amyloid-β	7.4 (*p* < 0.01)^*^	1.42	1.10–1.83
CSF p-tau	9.2 (*p* < 0.01)^*^	1.47	1.15–1.90
CSF t-tau	5.5 (*p* < 0.05)^*^	1.33	1.05–1.70
Hippocampal volume	4.8 (*p* < 0.05)^*^	1.31	1.03–1.67
Combination of CSF dichotomous amyloid-β and AD signature	19.4 (*p* < 0.0001)^**^	Aβ 3.0 ADsig 1.4	1.38–6.7 1.07–1.77

* *p* < 0.05,

** *p* < 0.005.

Finally, to begin to assess the specificity of the refined AD signature index measure we analyzed the relationships between CSF biomarkers and the raw AD signature measure (in millimeters) and the adjusted AD signature index measure (adjusted for thickness of the Aging signature regions as described in Section “Materials and Methods.” The adjusted AD signature index exhibited substantially stronger correlations (**Table 3**) with all CSF biomarkers relative to the raw AD signature, suggesting that this adjustment for “brain age” improves the specificity of this MRI biomarker for AD-related neurodegeneration.

**Table 3:** Relationships of CSF biomarkers to MRI biomarkers.

	Age	CSF t-tau	CSF p-tau	CSF amyloid-β
Aging signature	*r* = -0.38 (*p* < 0.001)	*r* = -0.05 NS	*r* = -0.04 NS	*r* = -0.06 NS
AD signature	*r* = -0.30 (*p* < 0.001)	*r* = -0.26 (*p* < 0.01)	*r* = -0.21 (*p* < 0.01)	*r* = -0.09 NS
Adjusted AD signature	*r* = 0.05 NS	*r* = -0.37 (*p* < 0.001)	*r* = -0.35 (*p* < 0.001)	*r* = 0.22 (*p* < 0.01)
Hippocampal volume	*r* = -0.19 (*p* < 0.05)	*r* = -0.04 NS	*r* = -0.03 NS	*r* = 0.05 NS

## DISCUSSION

When individuals are diagnosed with MCI, the two most pressing clinical questions relate to etiology and prognosis. We now have a growing armamentarium of biomarkers for AD and other neurodegenerative diseases, and reasonably mature diagnostic criteria for “MCI of the Alzheimer type” (Albert et al., 2011) which hinge on a typical clinical syndrome and the presence of one or more imaging or fluid biomarkers. In this analysis, we used ADNI data to test two major hypotheses in patients with MCI: (1) markers of amyloid and neurodegeneration provide distinct and complementary prognostic information over different time intervals, and that (2) evidence of neurodegeneration in amyloid-negative MCI individuals is useful prognostically. We found compelling support for both hypotheses.

For prediction of AD dementia in MCI, the AD signature cortical thickness biomarker performed better than hippocampal volume. Although CSF tau measures, also putative neurodegenerative biomarkers, were better than CSF amyloid-β at predicting dementia within 1 year, the AD signature was better than all CSF measures at prediction over this relatively short-term interval. CSF amyloid-β was superior to tau and AD signature at predicting dementia over 3 years. In an analysis examining the combined use of CSF and MRI measures, when CSF amyloid-β was dichotomized using previously published cutoff values and treated as a categorical variable, a Cox regression model indicated that both the AD signature MRI marker and the categorical CSF amyloid-β marker were useful in predicting time-to-event diagnosis of AD dementia. These results provide further support for emerging models of the pathophysiology of AD and demonstrate the utility of the combined use of these biomarkers at the prodromal stage of the illness (Jack et al., 2010; Landau et al., 2010; Vemuri et al., 2010).

A major novel contribution of the present study is the investigation of the prognostic utility of different biomarkers over intervals of varying times after the markers were obtained at baseline. To our knowledge, no prior study has explicitly examined separate follow-up intervals in MCI and measured the differential utility of amyloid vs. structural MRI markers. As we plan clinical trials of pharmacologic and non-pharmacologic interventions in MCI, it is critical not only to consider methods to homogenize the patient population for inclusion (e.g., requiring MCI patients to have cerebral amyloidosis for inclusion); it may also be valuable in some trial designs to use a marker of neurodegeneration to identify patients in whom progression to dementia is likely within a relatively short time interval, such as 1 year. Such a stratified design for inclusion might be valuable in that most such participants would be likely to decline substantially during a reasonable follow-up interval, thereby maximizing power to detect a beneficial effect of the intervention. Of course, it is also possible that in these more “aggressive” cases of prodromal AD a drug might be less efficacious than in more indolent forms of the disease, but that remains an open question.

These considerations are also becoming of greater relevance in clinical practice, particularly in light of recent FDA approval of the amyloid PET ligand florbetapir. Further, with the development of the above-described guidelines for incorporation of biomarkers into the assessment of MCI patients, it is likely that clinicians will be bringing these measures into their clinical practice for prognostication of MCI. The current work emphasizes that these tests may provide somewhat different information, which may have important implications for their value depending on the question that is being addressed. For example, MRI may be more valuable when interested in determining the likelihood of decline in the near future, which could influence life decisions that need to be made within that timeframe whereas the presence of amyloid may more definitively reflect the likelihood of progression, but have less value in predicting the timing.

It seems intuitive, based on current models of biomarkers of AD pathophysiology (Jack et al., 2013), that the presence of cerebral amyloidosis would be valuable for longer-term prognosis while an MRI-derived marker of neurodegeneration would demonstrate utility in shorter-term prognosis. As the individuals with MCI in this study were followed longitudinally, those with baseline cerebral amyloid progressed to dementia at a rate of about 15–20% per year, while only about 10–15% of those without baseline brain amyloid progressed to dementia after 3 years, most doing so within the first year of follow-up. Those who progressed to AD dementia at 3 years had baseline CSF amyloid-β levels that are similarly reduced to those who progressed at 1 year. This is consistent with models that suggest that amyloid deposition is an early feature of the disease that largely plateaus by the symptomatic stage of disease resulting in relatively poor resolution of disease state (i.e., proximity to dementia) at that stage (Villemagne et al., 2013). In contrast, the baseline MRI-derived AD signature measure of cortical thickness was substantially lower in individuals who progressed at 1 year than in those who progressed at 3 years (**Figure 2**). This indicates that once AD-related neurodegenerative cortical atrophy is prominent enough in MCI patients, further cognitive decline and loss of functional independence is imminent. Such a finding demonstrates the greater degree by which markers of neurodegeneration, particularly structural MRI measures, track disease state during symptomatic stages of disease.

The differences described here in temporal prediction and, ultimately, the complementary nature of biomarkers of cerebral amyloidosis with neurodegeneration are quite consistent with a number of recent studies in the literature exploring this issue. For example, Buchhave et al. (2012) recently described that while the presence of low CSF amyloid-β predicted conversion to AD in MCI patients, CSF p-tau status was associated with the timing of this conversion (abnormal: conversion in 0–5 years; normal: conversion in 5–10 years). Another group, also using the ADNI dataset, compared dichotomous measures of hippocampal atrophy, memory testing, and CSF total tau, p-tau, and amyloid-β in prediction of conversion. They found that median survival was generally shorter for neurodegenerative biomarkers while CSF amyloid-β had the longest median time before conversion (Heister et al., 2011). Further, using a FDG-PET “signature” of AD, similar to the structural one applied here, Landau et al. (2010) found that this measure was also superior to CSF amyloid-β for prediction of conversion in MCI patients with mean follow-up under 2 years. This group also described a tighter link between cognitive decline and cerebral amyloidosis, based on amyloid imaging, in asymptomatic individuals, but stronger association of decline with FDG-PET status in MCI (Landau et al., 2012). Thus, the current findings serve as additional support for the leading model of the proposed biomarker cascade (Jack et al., 2013), which has also found additional verification in longitudinal study of asymptomatic dominantly inherited AD mutation carriers (Bateman et al., 2012).

A variety of MRI measures have been proposed as potential biomarkers of neurodegeneration in early AD, both with regard to the identification of presumed atrophy consistent with AD and with regard to monitoring changes over time that indicate progression of neurodegeneration. Hippocampal volume is the most widely employed and discussed measure of this type, and while clearly informative, it is increasingly appearing to be less sensitive and specific than other measures such as regional cortical thickness. We have previously shown using receiver operating characteristic analyses that the AD signature measure is superior to hippocampal volume in discriminating individuals with prodromal AD who progress to dementia within 3 years from those who do not (Bakkour et al., 2009). Here we used logistic and Cox regression models to demonstrate the superiority of the AD signature over hippocampal volume in predicting progression to dementia in both amyloid-positive and amyloid-negative individuals with MCI. Nonetheless, it is worth noting that while not as strongly predictive of conversion as the AD signature, hippocampal volume still had predictive value in most of these analyses consistent with prior work using this measure (for review, see Frisoni et al., 2010). Further, much of the literature has applied cutoff values or categorical groupings of hippocampal volumes in similar analyses to those presented here, which may provide additional predictive power (Jack et al., 1999, 2010; Landau et al., 2010; Heister et al., 2011). Future work should explore optimized cutoffs for the AD signature and other structural measures, allowing for comparison of these measures in both continuous and dichotomous forms.

It is also important to note that we compared CSF molecular biomarkers on an individual basis, as opposed to the combination of these markers. However, it appears that a combination of these measures may further enhance prediction (Shaw et al., 2009; De Meyer et al., 2010). In particular, ratios of t-tau or p-tau to amyloid-β may improve prediction by incorporating both neurodegenerative and amyloid-based measures, akin to our finding that the combination of the AD signature and CSF amyloid-β produced the strongest model in the Cox regression analysis. Nonetheless, the current analysis was developed to specifically compare across these classes of biomarkers and, as such, we chose to keep the CSF measures uncoupled.

In the present study, we employed a novel approach to the calculation of our AD signature measure. In the past, we have generally not adjusted for age-related cortical atrophy, but in some analyses have simply corrected statistically for a participant’s chronological age. We recently reported on the cortical signature of normal aging (the “Aging signature”), describing a set of association and sensorimotor regions that undergo the most prominent loss of thickness in cognitively normal elderly adults compared to young adults (Bakkour et al., 2013). In the analyses here, we used the Aging signature set of regions to adjust for the “cortical age” of the individuals, creating an AD signature index, which represents the residual variance of the AD signature after accounting for variations in the Aging signature regional measurements. This corrects for the fact that some individuals may have thinner cortex in at least some of the regions vulnerable to AD simply as a result of more widespread cortical atrophy associated with normal aging, while those with thinner cortex in AD-vulnerable regions who have preserved thickness in Aging-vulnerable regions are much more likely to be exhibiting atrophy associated specifically with AD pathology. To our knowledge, this type of an adjustment of MRI biomarkers has not been performed previously. We are continuing to explore the strengths and weaknesses of this approach.

Finally, our analysis indicated that the MRI-derived AD signature biomarker was useful for predicting progression to dementia within 1 year in MCI participants with baseline CSF amyloid-β levels not low enough to meet typical cutoffs indicating cerebral amyloidosis. Even though the percentage of individuals who progressed to dementia in this subgroup was low (13%), the MRI marker was still useful for prediction in this short time interval. To interpret this finding, we have considered several possibilities. First, it is possible that these individuals have a non-Alzheimer pathology that is associated with atrophy in some of the same structures affected by AD. Although this consideration certainly seems reasonable when the structural MRI measure is of the hippocampus, since pathologies such as hippocampal sclerosis could be playing a role, it seems harder to reconcile with an MRI biomarker measuring a spatially distributed pattern of atrophy. We are currently examining the use of the AD signature marker in differential diagnosis of other neurodegenerative diseases, including frontotemporal dementias and Lewy body dementia; findings from this work will provide important data on the specificity of this marker in other neurodegenerative diseases. It is also possible that these individuals actually have underlying AD pathology but are “below the threshold” of amyloid pathology to meet current CSF cutoffs. This group could also be akin to previously reported cognitively normal and MCI individuals with evidence of AD-like neurodegeneration and negative amyloid status, which has been labeled suspected non-Alzheimer pathology (sNAP; Knopman et al., 2012; Petersen et al., 2013; Prestia et al., 2013). While debate continues regarding the underlying pathology in these individuals, Prestia et al. (2013) also similarly reported that such individuals with an MCI phenotype also had a high rate of conversion to clinical dementia. As they note, it is worth at least considering that these patients may require adaptation of the model of biomarker change used here, as has been recently discussed (Jack et al., 2013). It is also worth noting that the individuals in this group in the present analysis who progressed to dementia were diagnosed clinically with probable AD dementia rather than a non-AD dementia.

Limitations of the present study include the relatively short follow-up period and the small number of individuals who were amyloid-negative at baseline with adequate longitudinal follow-up data. Furthermore, a more broadly representative sample of individuals with MCI might be helpful to better determine whether these findings are generalizable to clinical practice. Nevertheless, we believe the results of the present analysis provide valuable insights about the use of biomarkers in an MCI sample likely to be similar to that considered for clinical trials of putative AD interventions.

## Conflict of Interest Statement

The authors declare that the research was conducted in the absence of any commercial or financial relationships that could be construed as a potential conflict of interest.

## Footnotes

1

2
